# CRISPR-Cas9 and Cas12a target site richness reflects genomic diversity in natural populations of *Anopheles gambiae* and *Aedes aegypti* mosquitoes

**DOI:** 10.1186/s12864-024-10597-4

**Published:** 2024-07-17

**Authors:** Travis C. Collier, Yoosook Lee, Derrick K. Mathias, Víctor López Del Amo

**Affiliations:** 1 Independent researcher, Vero Beach, FL 32962 USA; 2 Florida Medical Entomology Laboratory, Department of Entomology and Nematology, Institute of Food and Agricultural Sciences, University of Florida, Gainesville, FL 32962 USA; 3 Center for Infectious Diseases, Department of Epidemiology, Human Genetics, and Environmental Sciences, School of Public Health, University of Texas Health Science Center, Houston, TX 77030 USA

**Keywords:** CRISPR, Genome editing, Cas9, Cas12a, Mosquitoes, Malaria vector, Dengue vector, Genetic polymorphisms

## Abstract

Due to limitations in conventional disease vector control strategies including the rise of insecticide resistance in natural populations of mosquitoes, genetic control strategies using CRISPR gene drive systems have been under serious consideration. The identification of CRISPR target sites in mosquito populations is a key aspect for developing efficient genetic vector control strategies. While genome-wide Cas9 target sites have been explored in mosquitoes, a precise evaluation of target sites focused on coding sequence (CDS) is lacking. Additionally, target site polymorphisms have not been characterized for other nucleases such as Cas12a, which require a different DNA recognition site (PAM) and would expand the accessibility of mosquito genomes for genetic engineering. We undertook a comprehensive analysis of potential target sites for both Cas9 and Cas12a nucleases within the genomes of natural populations of *Anopheles gambiae* and *Aedes aegypti* from multiple continents. We demonstrate that using two nucleases increases the number of targets per gene. Also, we identified differences in nucleotide diversity between North American and African *Aedes* populations, impacting the abundance of good target sites with a minimal degree of polymorphisms that can affect the binding of gRNA. Lastly, we screened for gRNAs targeting sex-determination genes that could be widely applicable for developing field genetic control strategies. Overall, this work highlights the utility of employing both Cas9 and Cas12a nucleases and underscores the importance of designing universal genetic strategies adaptable to diverse mosquito populations.

**Supplementary Information:**

The online version contains supplementary material available at 10.1186/s12864-024-10597-4.

## Introduction

The advent of **c**lustered **r**egularly **i**nterspaced **s**hort **p**alindromic **r**epeats (CRISPR) has opened new possibilities for genetic control for various insect disease vectors [1–9]. Multiple CRISPR-based gene-engineering approaches, such as gene drives, show particular promise for controlling insect populations [10]. CRISPR gene drives propagate alleles of interest into a specific population via self-replicating genetic elements, either spreading beneficial traits to confer pathogen resistance for population modification or deleterious mutations targeting sex-determination genes for population suppression [10].

Population modification approaches have been developed in various disease vectors impacting public health including *Anopheles gambiae* (*Agam*), *Anopheles coluzzii* (*Acol*), *Anopheles stephensi* (*Aste*), and *Aedes aegypti* (*Aaeg*) [2–4, 6, 11]. Population suppression strategies have been developed in *Agam* [5, 12, 13]. While mosquitoes are responsible for 350–650 million cases of malaria and dengue alone [14], current methods such as insecticides and source reduction often fall short due to insecticide resistance and cryptic larval habitats [15]. The highly invasive nature of *Aaeg* as well as increasing numbers of new mosquito introductions into non-native habitats further supports the need for alternative approaches including CRISPR-based genetic control strategies. Indeed, the integration of genetic control methods into routine integrated pest management operations will be a paradigm shift in vector control programs for reducing the impact of diseases such as malaria or dengue transmitted by these mosquitoes.

While CRISPR gene drives have shown great promise at managing *Anopheles* and *Aedes* mosquitoes under laboratory conditions, their field implementation remains unclear since all experiments have been performed under laboratory conditions, and laboratory colonies could behave differently compared to natural populations [16, 17]. Indeed, the establishment of laboratory colonies imposes strong selection and a population bottleneck leading to lower levels of genetic diversity compared to natural populations [18, 19]. This is highly relevant as CRISPR-based approaches rely on DNA recognition sites for targeting and achieving precise genome editing [20, 21]. Therefore, DNA polymorphisms existing in natural populations could lead to the development of resistance and undermine the efficacy of a deployed genetic control strategy.

Recent works have highlighted this issue by gathering genomic data from natural populations where meaningful levels of genetic diversity have been observed in *Anopheles* and *Aedes* mosquitoes [22–24]. Furthermore, CRISPR gene drives can introduce insertions or deletions (indels), known as resistant alleles, when the wildtype allele is not efficiently replaced. This could favor the accumulation of these resistant alleles, thereby reducing the spread of the gene drive necessary for population engineering [25, 26].

For all the reasons mentioned above, a detailed evaluation of the polymorphisms within natural populations is critical for designing broadly applicable guide RNAs (gRNAs). This will guide researchers developing gene drives and other CRISPR-based systems in the laboratory to consider highly conserved regions that will enhance the likelihood that their constructs will be efficient in controlling natural populations. This will in turn, improve the chance of success in field trials and improve sustainability when deployed in real-world settings.

The richness of target sites considering sequenced genomes from natural populations was described for *Agam/col* and *Aaeg* [27]. This study, however, included non-coding regions, which are not typically used in CRISPR gene drives strategies. Other resources, such as the elegant CRISPR GuideExpress, support the identification of pre-computed CRISPR gRNA designs for mosquitoes [28, 29]. Yet, this software provides gRNA candidates with predictable efficiency only for *Anopheles* species employing Cas9. While all current CRISPR gene drives for vector control rely on the Cas9 nuclease [10], we have recently described a proof-of-concept gene drive system using Cas12a nuclease in *Drosophila melanogaster* [30], which opens a new avenue towards next-generation gene drive systems utilizing a different nuclease while deserving further analysis in mosquitoes. For example, Cas12a requires a TTTN DNA recognition PAM site (where N is any nucleotide) for targeting, as opposed to the NGG PAM of Cas9. Therefore, a Cas12a system could target T-rich genomic regions currently inaccessible with Cas9. In summary, CRISPR target sites studies utilizing genomes from wild *Anopheles* and *Aedes* populations are needed.

In this work, we perform an analysis of the two principal human disease vectors *Agam*, and *Aeag* for the presence of CRISPR-Cas9 and CRISPR-Cas12a target sites using genomes from natural mosquito populations. Specifically, we screen for available gRNA designs within coding region sequences (CDS). We first evaluate potential target sites using the reference genome generated from laboratory colonies and then apply a subset of filters to identify CRISPR designs that could be applied both in the laboratory and potential field implementations.

We identified hundreds of thousands of CRISPR target sites for both Cas9 and Cas12a nucleases; however, only ~ 2% of target sites passed our filtering process when considering polymorphisms present in natural populations. We also revealed differences between *Aaeg* from populations in North America and Africa in target site abundance due to nucleotide diversity. Lastly, we searched for suitable gRNAs targeting genes for population suppression and identified new gRNAs that could be translated into field applications. Our analysis concludes that polymorphisms from natural populations will not constitute an obstacle to the efficient implementation of CRISPR-based approaches while highlighting the importance of carefully revising the gRNAs employed in laboratory settings and their suitability for field implementation.

## Results

### Identifying potential CRISPR-Cas9 and Cas12a target sites

For exploring all potential available target sites of Cas9 or Cas12a in each species’ genomes, we used two different target site criteria for each nuclease. For Cas9, we define potential target sites as 23 bp nucleotides, where the DNA recognition site (PAM sequence) is a ‘NGG’ nucleotide sequence at the 3′-end of the gRNA sequence, located within the coding sequence (CDS) of any gene. For Cas12a, we define potential target sites as 27 bp nucleotides with the DNA recognition site (PAM sequence) being a ‘TTTN’ nucleotide sequence at the 5′-end of the gRNA sequence located within CDS regions.

Additionally, we added more constraints in determining potential targets using parameters that affected gene drives’ efficiency in our past experience. We limited our search for potential target sites for both Cas9 and Cas12a to coding sequence (CDS) regions with a GC content ranging from 40 to 80%. Since three hydrogen bonds are formed for a G: C base pair instead of two for a A: T pairing, higher GC content increases the gRNA-DNA interaction [31], and are expected to increase gene editing efficiency of the CRISPR system. Also, we excluded targets having potential off-target effects elsewhere in the genome with close matches (< 4 mismatches). Lastly, we have discarded any gRNA sequence with a CHOPCHOP efficiency score lower than 50. CHOPCHOP is a software that predicts gRNA efficiency when designing CRISPR strategies [32], and based on our past experience building gene drives, gRNAs with a CHOPCHOP efficiency score lower than 50 reduces the efficacy of gene drives.

The off-target filter had the most significant effect in reducing Cas9 target sites (32.49% pass), while CHOPCHOP score efficiency filter had the largest impact in reducing Cas12a (31.73% pass) (Supplementary Table 1). This correlates with a previous study demonstrating that off-target sequence recognition and cleavage are lower in Cas12a compared to Cas9 [33]. While the efficiency score is based on machine learning algorithms [34], actual gene drive efficiency may deviate from this expectation in vivo. Recent studies reported a wide range of gRNA activities using Cas12a in *Drosophila melanogaster* in comparison with Cas9 [30, 35]. The functionality of Cas12a in mosquitoes still needs to be demonstrated. Since the amount of published works using Cas12a in insects is still limited, future CRISPR-Cas12a designs for vector control will help better understand the dynamics of gene drive systems using alternative nucleases like Cas12a.

After identifying potential targets in the CDS region that passed all the requirements listed above, we applied additional filters to identify “good targets”. We define good targets as those that hold 99% chance of matching the genomes accounting for genetic polymorphisms within natural populations. The total number of target sites was estimated by screening the reference genomes of *An. gambiae* (AgamP4) and *Ae. aegypti* (AaegL5) [23]. We used genomic data from field samples totalling 182 and 284 for *Agam* and *Aaeg* mosquitoes, respectively.

We have identified 3,932,873 and 3,665,000 Cas9 targets in AgamP4 and AaegL4, respectively. After applying all the aforementioned filters, we observed 13.13% (516,241) for AgamP4 and 10.35% (379,147) for AaegL5 potential target sites. Furthermore, we detected 2.92% (114,669) and 2.1% (76,961) good targets, when accounting for polymorphisms found in natural populations (Table 1).

For Cas12a, we have identified 1,535,035 (AgamP4) and 1,695,992 (AaegL5) targets before filtering, which is lower compared to Cas9. Then, we observed 12.3% (188,834) for AgamP4 and 12.48% (265,419) for AaegL4 potential target sites after applying the filters. Lastly, we detected 2.47% (37,969) and 2.52% (53,494) good targets (Table 1). While ~ 2–3% good target sites may seem a concern, it is important to keep in mind that these percentages still represent hundreds of thousands of gRNA sequences that could be designed for both Cas9 and Cas12a nucleases to efficiently target the mosquito genomes.

**Table 1:**
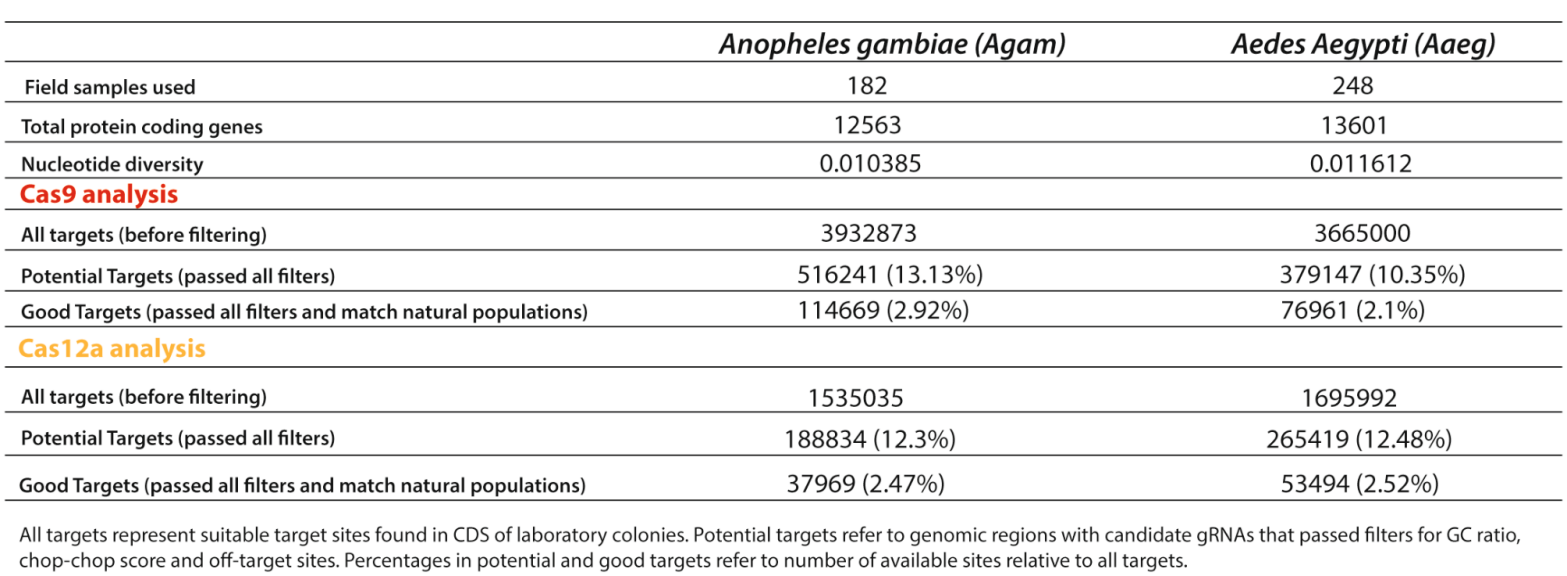
Predicted target sites fo Cas9 and Cas12a nucleases in mosquito genomes

### Differences between North America and Africa *Aedes* populations

While the natural distribution of African malaria vector *An. gambiae* is confined to Africa, invasive arbovirus vector *Ae. aegypti* has global distribution spanning multiple continents [36]. *Aedes aegypti* originated from Africa and the leading hypothesis suspects that *Ae. aegypti* is introduced to the Americas during the slave trade period 400–550 years ago. With recent publications of genome data originating from various populations from Africa and the Americas [27, 37, 38] as well as new data generated from this study, we can examine the continental differences among mosquito populations.

While all the field samples (182) from *Agam* are originally from Africa, the *Aaeg* field samples (248) are either from North America (N. America) or Africa. Specifically, 156 field samples were from N. America and 89 from Africa. Interestingly, the African samples displayed higher nucleotide diversity in coding regions despite the fewer samples than the N. American mosquito samples (Table 2). This observation is in line with previous works with genetic data where higher nucleotide diversity in African mosquito populations was also observed [24, 39–41]. These observations translate into significant differences in the number of suitable targets; N. American and African mosquito populations showed 165,055 (4.5%) and 49,579 (1.35%) good targets when using Cas9, respectively. Similarly, Cas12a-based good targets displayed 106,993 (5.03%) and 36,701 (1.73%) good targets when comparing *Aedes* mosquito field samples from N. America and Africa, respectively. This suggests that lower nucleotide diversity would involve higher conservation within target sites, providing more opportunities to identify suitable genome areas for gRNA designs in N. America *Aedes* mosquito populations. Indeed, these observations show the importance of identifying gRNA sequences conserved across all populations to build universal genetic strategies that can be implemented worldwide.

**Table 2:**
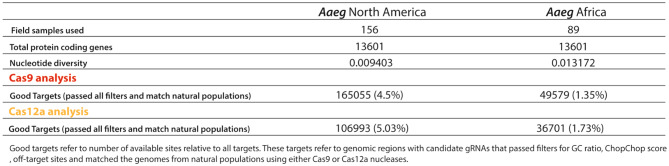
Predicted target sites fo Cas9 and Cas12a nucleases comparing Aaeg mosquitoes collected in North America or Africa

### Increased number of good targets per gene with Cas9 and Cas12a nucleases

Different CRISPR-based genetic strategies require multiple target sites. For example, multiplexing gene drive approaches seeking to propagate engineered alleles into a target population utilize several gRNAs to target nearby locations within the same gene to boost DNA homology-directed repair (HDR) [42–44]. Other strategies, such as cleave and rescue (ClvR), disrupt an essential gene while providing a second version of the essential locus resistant to cleavage elsewhere in the genome, and this facilitates the increased frequency of ClvR individuals as insects lacking the ClvR transgene die [45, 46]. Also, precision-guided sterile genetic insect technique (pgSIT) seeks to produce sterile males for mass release by targeting sex-determination genes employing multiple gRNAs targeting the same locus to maximize efficient gene disruption [7, 47].

**Fig. 1:**
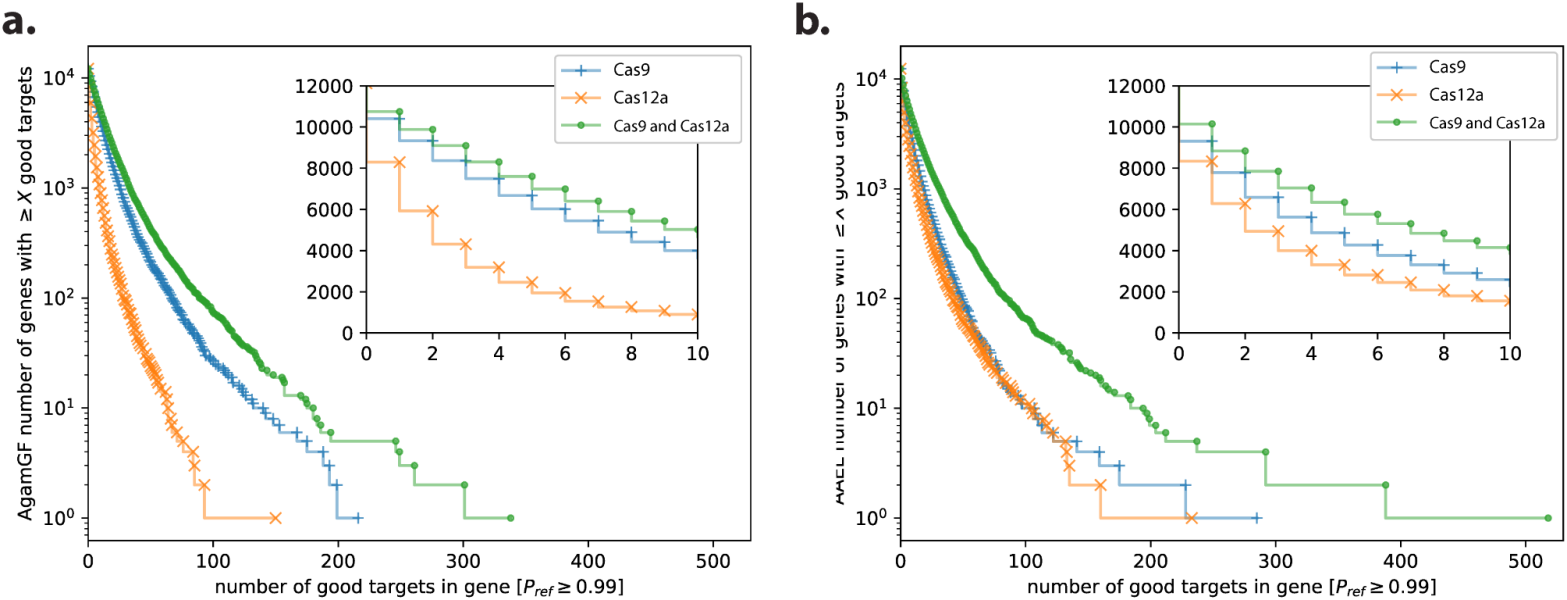
Number of good targets per gene for Cas9, Cas12a and combined. (**a**) Number of good targets in *Anopheles* populations are depicted. An inset zooming is provided to highlight number of genes with 0 to 10 available targets. (**b**) Number of good targets in *Aedes* populations are depicted. An inset zooming to highlight number of genes with 0 to 10 available targets is provided. P_ref_ ≥ 0.99 in the X axis indicates that all gRNA sequences should match 99% of the natural population genomes

Since different strategies would benefit from having multiple target sites, we wondered about the number of genes with multiple target sites employing Cas9 and Cas12a in both *Agam* and *Aaeg* mosquitoes. First, we found that the number of genes with multiple good target sites is higher when using the Cas9 nuclease compared to the Cas12a nuclease in *Agam* mosquitoes (Fig. 1a). Most importantly, we found that having two nucleases increased the number of genes with over 10 good target sites, with more than 6,000 genes showing this capability when both Cas9 and Cas12a were employed together (Fig. 1a). In *Aedes* mosquitoes, Cas9 and Cas12a good target sites were more similar in numbers compared to *Anopheles* mosquitoes (Fig. 1b). This could be because more than 40% of the *Aedes* genome is composed of repeated sequences like transposable elements, and these elements have shown high AT-richness [48, 49], which would favor the number of Cas12a sites requiring a ‘TTTN’ PAM for DNA targeting. To confirm this idea, we have calculated the GC (%) content from CDSs only for both *Anopheles* and *Aedes*. Considering the reference genomes, these mosquitoes present 56.5% and 49.8% GC content, respectively (see ‘Availability of data and material’ section for more information), confirming that *Aedes* mosquitoes display higher AT richness in the CDS compared to *Anopheles*. As we observed with *Anopheles* mosquitoes, having both nucleases is beneficial for increasing the number of target sites in *Aedes* since more than 4,000 loci displayed 10 target sites per gene when both nucleases were combined (Fig. 1b).

### Targeting genes with both Cas9 and Cas12a nucleases for population suppression strategies

While our analysis highlights the benefits of having two nucleases to increase the number of target sites, we wonder if these observations could be translated to specific genes used in population suppression strategies.

**Fig. 2:**
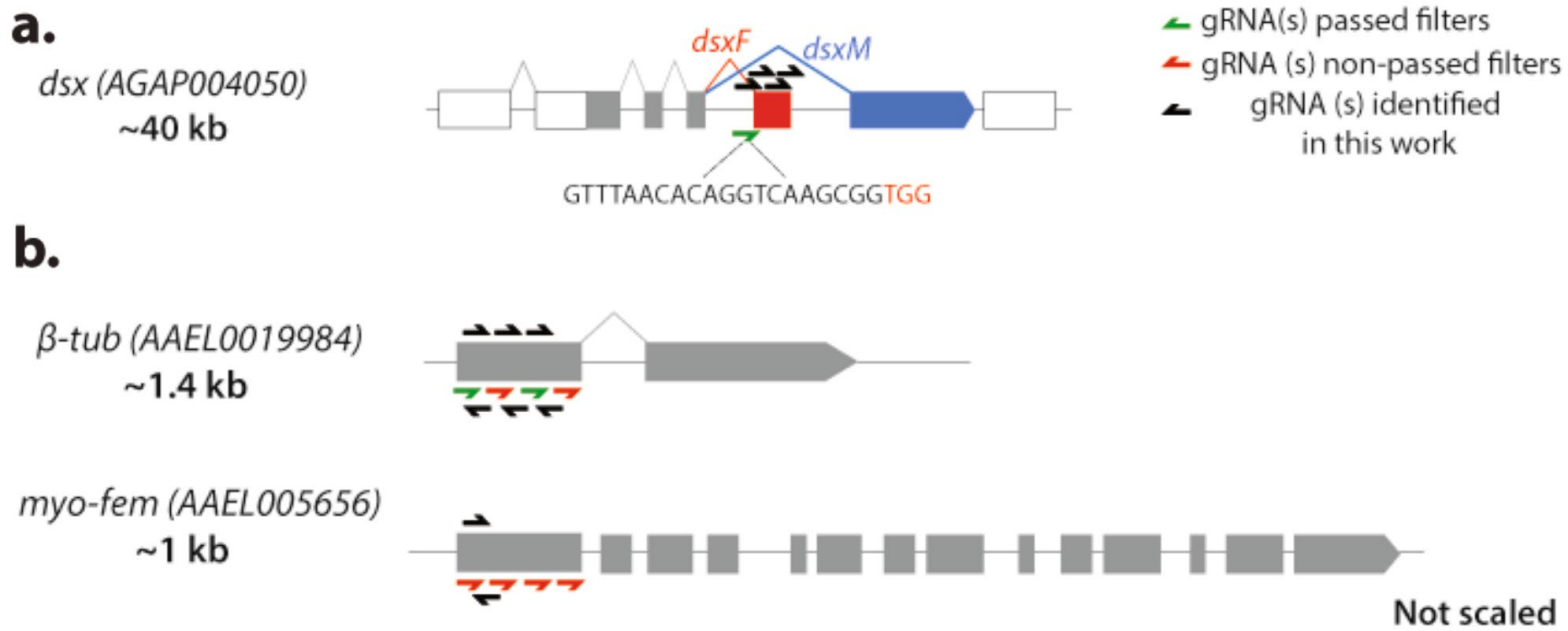
Analysis of CRISPR designs from previous works in population suppression strategies. (**a**) The gRNA designed in gene drives for population suppression, which did pass our filtering evaluation, is depicted. Coding exons marked in gray. Red exon indicated female-specific transcript while blue indicates male-specific exon (**b**) The gRNAs employed in pgSIT strategies for population suppression are highlighted. Coding exons are represented in gray. gRNAs in red indicate sequences that did not pass our filters. Green gRNAs indicate sequences that appeared in our target sites analysis. gRNAs in black indicate new gRNA sequences identified in this work. All gRNA from these studies and the additional ones found in our studies are available in Supplementary Table 2

For example, several elegant works using gene drives and targeting the double-sex gene in *Anopheles gambiae* have demonstrated their efficiency at suppressing mosquito populations under laboratory conditions [5, 12, 13]. This design targets exon 5, which is only required for female development (Fig. 2a). Here, each time a gene drive male mates with a wildtype female, it will produce viable males carrying the gene drive and intersex (females with male traits) sterile females. Therefore, the accumulation of infertile females over generations leads to the elimination of mosquito individuals [5, 12]. All these works employ the same gRNA sequence targeting the intron 4-exon 5 junction (Fig. 2a), and while it showed great efficiency, the authors state that 3% of the wild populations contain a polymorphism within the gRNA sequence, which could compromise the efficiency of this gene drive prototype in the field. Importantly, our filtered analysis captured the gRNA employed in previous works using wild populations and identified four additional gRNAs (2 for Cas9 and 2 for Cas12a) that could be used for gene drive purposes (Supplementary Table 2), as discussed below.

Regarding *Aedes* mosquitoes, gene drives for population modification have been described [6, 8, 50], yet no gene drive system has demonstrated efficient introgression in population suppression settings. Instead, the sophisticated precise guided sterile insect technique (pgSIT) demonstrated its efficiency at suppressing mosquito populations under laboratory settings. Here, sterile males are generated via a genetic cross of two transgenic lines and released for reducing population numbers: a Cas9 strain and a line containing a set of guide-RNAs (gRNAs) targeting genes involved in male sterility and female lethality or infertility, which, when genetically combined, produce only sterile male progeny [47]. Specifically, *β-Tubulin* (*β-Tub*) and *myosin heavy chain* (*myo-fem*) loci have been targeted in mosquitoes since their disruption causes mutant male flies to be sterile and selective flight impairment of females [7, 51], respectively.

In these works, 4 gRNAs were designed to target the *β-Tub* locus (Fig. 2b) and only two of them passed through the filtering that we applied in this work. Most importantly, our analysis identified three additional gRNAs using Cas9 and three if employing Cas12a nuclease targeting the same exon, which passed all the filters and therefore should match the field genomes (Supplementary Table 2). Additionally, the gRNAs targeting the *myo-fem* were not able to pass our filters, suggesting these sequences would not be ideal for field population applications (Fig. 2b). Yet, our analysis revealed that one gRNA could be designed using Cas9, and Cas12a would provide one additional target site if editing the same exon (Supplementary Table 2). It is important to note that previous works edited the *β-Tub* and *myo-fem* first exon [7, 51], yet, other gRNAs targeting different exons could be tested. We are aware the purpose of the pgSIT strategy is to release males that represent a dead end and do not need to target the genome from wildtype populations as gene drives. Yet, if possible, we believe that an ideal scenario for future field interventions would imply the generation of sterile males using field population colonies. Here, the gRNA design would require more specificity to match the genome’s population.

## Discussion

This work shows the amount of realistic available target sites for mosquito genome editing using Cas9 as well as Cas12a. Importantly, CRISPR target sites were analyzed using reference genomes from laboratory colonies and mosquito genomes from natural populations. We added samples from different locations to have a broad geographic span. We show that from all potential targets identified in laboratory colonies, only ~ 2% passed all our filters including GC content, off-targets, CHOPCHOP efficiency score analysis and considering genomes from natural mosquito populations.

We observed that Cas9 displayed more target sites compared to Cas12a in both *Anopheles* and *Aedes* populations, and this could be understood by the more restricted requirements in terms of gRNA length and PAM sequence by the Cas12a. While the optimal gRNA length for Cas9 is 20 nucleotides along with the ‘NGG’ trinucleotide PAM sequence [20], the Cas12a relies on a 23 nucleotide gRNA sequence and a ‘TTTN’ PAM sequence [21], which could reduce our chances to find a suitable genomic region. However, it is important to note that the Cas12a requirements will allow it to target AT-rich regions that could not be edited by the Cas9 nuclease needing a ‘NGG’ sequence region. In line with this, we observed that having two nucleases boosted the number of good targets per gene, and this will favour the design of different strategies such as multiplexing or pgSIT strategies, which employ multiple gRNAs to target the same locus [6, 7, 42].

Interestingly, we also observed differences in target sites when separating the *Aedes* natural population genomes by geographic areas. We observed that *Aedes* populations from N. America displayed a higher number of target sites compared to *Aedes* mosquitoes from Africa. This can be explained by differences in nucleotide diversity between these two populations, and support the demographic history that mosquitoes from Africa preceded the established *Aedes* mosquitoes in America [52]. Even more important, it highlights the necessity of carefully designing genetic strategies that could be applied for mosquito populations inhabiting different geographical areas. Another observation from this point is that the variability between these two populations could enable the design of a drive that selectively targets one population over another. This capability could be particularly desirable, especially in targeting invasive populations of a species without inadvertently affecting native populations.

Also, we analyzed the CRISPR-Cas9 designs from previous works targeting *Anopheles* [12] and *Aedes* [7] mosquitoes for population suppression. In the first case, we observed that the gRNA designed in gene drive settings also appeared in our CRISPR-Cas9 target sites scanning. Yet, the authors recognized that 3% of the wild populations contained a polymorphism within the target site [12]. While the authors demonstrated CRISPR in vitro activity at this polymorphism allele, it is important to note that genome editing levels can be higher in vitro than in vivo [53]. Importantly, we have identified four additional gRNAs using either Cas9 (2 sites) or Cas12a (2 sites). Although the gene drive efficiency in this work was extremely high, the Cas9 gRNAs identified in this study could be combined with the previously tested and built next-generation strategies through multiplexing approaches. With this strategy, when two gRNAs are integrated into the same gene drive element, multiple cuts are introduced into the DNA to potentially improve gene drive efficiency [42, 43], and this could bypass potential resistance development concerns. Also, the current Cas9 system could be combined with the Cas12a gRNA to build double gene drive systems where two independent gene drive elements spread simultaneously, as we recently described [30]. Lastly, we evaluated previous CRISPR-Cas9 designs in pgSIT strategies using *Aedes* mosquitoes; this system was highly successful in the laboratory at suppressing mosquito populations by producing male sterility disrupting *β-Tub* and female flight and blood-consumption impairment. As mentioned above, we know that released pgSIT males in potential field applications represent a dead end, and genome editing of the mosquitoes in the field is unnecessary. However, we believe that an ideal scenario for future field interventions would involve generating sterile males using field population colonies, if possible. In this context, the gRNA design would need to be more specific to effectively target the population’s genome.

Gene drive systems for population modification seeking to spread beneficial traits (i.e., anti-malaria effector cassettes) also hold promise. In fact, *Anopheles* gene drive approaches targeting pigmentation loci have shown full introgression of the engineered alleles carrying antiparasite effector genes [2, 4], others expressed antimalarial antibodies targeting Lipophorin or Saglin loci while displaying spreading of the genetic elements in laboratory settings [11]. Lastly, a relevant work expressed antimicrobial peptides that could block parasite development in the mosquito guts if linked to a gene drive system [54]. Since population modification approaches rely on the expression of antibody cassettes that are propagated to protect mosquitoes against the pathogens, the spectrum of genes to target is more flexible, as long as they do not generate any fitness cost [1, 55]. Instead, population suppression strategies rely on sex-determination genes, which are limited within the genome, and therefore require a more profound analysis. Also, gene drives can generate indels in both population modification and suppression strategies, yet, suppression strategies are in principle less tolerant to the formation of these resistant alleles, which could represent an obstacle for gene drive efficiency [56–60]. While we did not explore gRNAs employed in previous population modification strategies, a thorough analysis is also recommended in these approaches before field implementation.

Overall, the study highlights the importance of comprehensive genomic analysis and population-specific considerations when designing CRISPR-based interventions for mosquito population control. By identifying target sites with high specificity and efficiency, researchers can develop more effective and tailored approaches for combating vector-borne diseases.

## Methods

The same methods were used to search for potential CRISPR-Cas9 and CRISPR-Cas12a target sites for *Agam*, *Acol*, and *Aaeg* genome as in Schmidt et al. 2020. Briefly, potential target sites were searched using command line version of CHOPCHOP version 3 with Python version 2.7.15, applying default setting and using efficiency scoring models ‘XU_2015’ for Cas9 and ‘KIM_2018’ for Cpf1.

CHOPCHOP was run to identify target sites for every transcript within each reference genome, comprising 12,562 protein-coding-genes in AgamP4.11 and 13,601 in AaegL5.1. Duplicates were removed, and the targets were then filtered to retain only ones where the cleavage site(s) fell within a CDS. The output was filtered for targets that show no off-target sites with less than four mismatches to the original sequence, a GC content between 40% and 80%, and an efficiency score of at least 0.5 (0 to 1 scale). Target sites passing all these filters are denoted “potential target sites.”

We further filtered target sites based on the frequency of polymorphism within the gRNA matching sequence found in natural populations. Population variation data from previous studies [22, 24, 27, 38, 61, 62] (182 Agam and 212 Aaeg samples) with the addition of 36Aaeg samples were used for this analysis. The probability of a polymorphism (P_ref_) for each target was calculated assuming each variant is independent, and a threshold value of P_ref_≥0.99 corresponding to a 1% chance of the target site containing a variation was used for filtering. Potential target sites passing this filter are denoted “good target sites”. Filtering and collation of results was conducted using python 3.10.8 in jupyter lab 4.0.6 with pandas 1.5.2 [63] and scikit-allel 1.3.5 [64].

## Electronic supplementary material

Below is the link to the electronic supplementary material.

Supplementary Material 1

Supplementary Material 2

Supplementary Material 3

## Data Availability

Sequence data sources are from [22, 24, 27, 38, 65]. Additional 36 Aaeg genome data can be accessed from NCBI BioProject PRJNA1013434 and PRJNA1090933. Sample metadata for Aaeg is provided in Supplementary Data 3. Data processing scripts, small data files, gRNA sequences identified in this study as ‘good targets,’ and overall (%) GC content for both the whole genome and only the CDSs based on the reference sequences are available on GitHub with the identifier: travc/Agam-Aaeg-CRISPR-Cas9-and-Cas12a-Target-Sites. Any additional data that support the findings of this study are available from the corresponding author upon reasonable request.
